# Distribution of extended-spectrum β-lactamases (ESBLs) among *Salmonella* serogroups isolated from pediatric patients

**Published:** 2018-10

**Authors:** Reza Ranjbar, Mehrdad Ardashiri, Sakineh Samadi, Davoud Afshar

**Affiliations:** 1 Molecular Biology Research Center, Systems Biology and Poisonings Institute, Baqiyatallah University of Medical Sciences, Tehran, Iran; 2 Department of Microbiology, Islamic Azad University, Damghan Branch, Damghan, Iran; 3 Department of Microbiology and Virology, School of Medicine, Zanjan University of Medical Sciences, Zanjan, Iran

**Keywords:** *Salmonella*, Extended-spectrum beta-lactamases, Ciprofloxacin resistance

## Abstract

**Background and Objectives:**

Extended-spectrum β-lactamases (ESBLs) and fluoroquinolones are generally used to treat invasive *Salmonella* infections, but emergence of antibiotic-resistant strains are increasing worldwide. This study was aimed to investigate the distribution of ESBLs among *Salmonella* serogroups isolated from pediatric patients in Tehran, Iran.

**Materials and Methods:**

The study included all *Salmonella* isolates recovered from pediatric patients admitted to Children’s Medical Center, Tehran, Iran during 2015–2016. Bacterial isolation and identification were performed by standard biochemical and agglutination tests. Antimicrobial susceptibility testing was done according to the Clinical and Laboratory Standards Institute (CLSI). Polymerase chain reaction was used to identify the genetic determinants responsible for ESBL phenotypes.

**Results:**

A total of 138 *S. enterica* serovars were isolated from stool specimens, including serogroup A (1), serogroup B (18), serogroup C (41) and serogroup D (78). Forty isolates out of 138 *Salmonella* strains had shown ESBL-positive phenotype. All ESBL-positive isolates showed multiple resistant phenotype. Resistance to more than 3 antimicrobial agents was observed among ESBL-positive strains. The frequency of *Salmonella* strains carrying the *bla*_CTX_, *bla*_TEM_ and *bla*_SHV_ genes was 17 (12.3%), 40 (29.9%) and 4 (2.89%) respectively.

**Conclusion:**

The high rates of ESBLs positive-*Salmonella* strains recovered from pediatric patients is alarming and indicates a necessity to substitute the cephalosporins with a proper alternative.

## INTRODUCTION

Gastroenteritis, also known as infectious diarrhea, accounts for the most important health problems worldwide, particularly in developing countries (1, 2). *Salmonella* is considered as one of the most common bacterial causes of acute gastroenteritis and food-borne infections worldwide (3). *Salmonella* gastroenteritis is usually a self-limiting disease. *Salmonella* species are prevalent throughout the world including Iran (4).

Extensive use of antibiotics in humans leads to antibiotic resistance among some bacterial species (5). Application of antibiotics in animal feeds, especially in poultry industry, also led to increased antibiotics resistance (6). *Salmonella enterica* serovars are associated with poultry settings and therefore, the consumption of their products may increase the risk of antibiotic-resistance distribution to humans (7). The resistant strains are now common worldwide and it is believed that they acquire their resistance in the animal hosts (8).

Antibiotic resistance among *Salmonella enterica* serovars has been increasing (9, 10) and existence of isolates with resistance to several antibiotics is also a concern in treatment of salmonellosis. Resistance to antibiotics due to production of β-lactamase enzymes is common among *Salmonella* strains and other gram negative bacilli. Recent studies show that *Salmonella enterica* strains isolated from different countries, carry the extended-spectrum β-lactamases (ESBLs) such as CTX-M, SHV, TEM and ACC-1 enzymes (11, 12).

There is limited data about the prevalence of *Salmonella* producing ESBLs in Iran (13, 14), however, it seems to be more than what is reported in the published literatures. The aim of the present study was to characterize the β-lactamase–producing *Salmonella* isolates recovered from pediatric patients with diarrhea in Tehran, Iran over a 2-year period.

## MATERIALS AND METHODS

### Bacterial culture and isolation.

The study included all *Salmonella* isolates recovered from pediatrics patients admitted to Children’s Medical Center, Tehran, Iran from Jan. 2015 to Dec. 2016. The stool specimens were transferred into Selenite-F Broth and incubated at 37°C for 6 h. These cultures were again sub-cultured on *Salmonella*-Shigella agar and Bismuth sulphite agar (Merck, Germany) and finally, single colonies were identified using standard biochemical tests (15). Then, *Salmonella* isolates were serogrouped by commercial typing anti-sera.

### Antibiotic susceptibility testing and ESBL screening.

Antibiotic susceptibility was determined according to the Clinical and Laboratory Standard Institute (CLSI) standards using antibiotic discs (MAST UK). The antibiotics selected for the panel were the following: ampicillin (AMP 10 μg), ciprofloxacin (CIP, 5 μg), ceftriaxone (CRO, 30 μg), cefotaxim (CTX, 30 μg) and ceftazidime (CAZ 30 μg). Briefly, bacterial suspensions were provided from overnight cultures, adjusted to the 0.5 McFarland turbidity standard and then, the organisms were evenly spread on the surface of a 10×150 mm Muller Hinton agar (Difco, USA) plate using a cotton swab. After about 15 min, the disks were applied to the plates and incubated at 37°C for 18h. Finally, the diameter of the inhibition zone was measured using a ruler.

The double-disk synergy method was used by applying of ceftriaxone (CRO, 30 μg), cefotaxim (CTX, 30 μg) and ceftazidime (CAZ 30 μg) next to (25 mm) a disc of augmentin (amoxicillin 20 μg + clavulanate 10 μg). The zone of inhibition greater than 5 mm of cephalosporin toward the augmentin disc was interpreted as positive for ESBL production.

### pCR.

ESBL positive isolates were cultured on LB broth and incubated at 37°C for 24 hr. The cultures were centrifuged at 9000 RPM for 3 min and genomic DNA was extracted using QIAamp DNA Mini Kit (Qiagen, Germany) according to the manufacturer’s instructions. The concentration of extracted DNA was calculated by an ND-1000 spectrophotometer (Nano Drop, Wilmington, DE, USA).

For each *bla*_TEM_, *bla*_SHV_ and *bla*_CTX-M_ gene, polymerase chain reaction was carried out in a final 25 ul reaction mixture containing 12.5 μL of 2×PCR Master Mix, 10 pmol of both forward and reverse primers (Bioneer, Korea) (Table 1) and 50 ng DNA.

**Table 1.** Primers used in this study.

**Target gene**	**Primer name**	**Primer sequence (5′→3′)**	**Amplicon size (bp)**	**Reference**
*bla*_TEM_	TEM-F	ATGAGTATTCAACATTTCCG	867	(16)
	TEM-R	CTGACAGTTACCAATGCTTA		
*bla*_SHV_	SHV-F	TTAACTCCCTGTTAGCCA	796	(17)
	SHV-R	GATTGCTGATTTCGCCC		
*bla*_CTX-M_	CTX-F	CGCTTTGCGATGTGCAG	550	(17)
	CTX-R	ACCGCGATATCGTTGGT		

The PCR was run at the following temperatures cycles: initial denaturation at 94°C, 5 min; 30 cycles of 94°C for 30 s, [60°C for 45 s (CTX-M), 54°C for 30 s (SHV), 52.2°C for 60 s (TEM)], and 72°C for 1 min; and final extension at 72°C for 10 min using a thermocycler (Eppendorf (W/V) agarose gel, stained with a DNA Safe Stain (CinnaGen, Iran) and finally visualized under a gel documentation system (Bio-Rad, Germany).

## RESULTS

A total of 2,200 stool specimens were obtained from patients with diarrhea. One hundred- thirty-eight *S. enterica* serovars were isolated from stool specimens, that included serogroup A (n=1), serogroup B (n=18), serogroup C (n=41) and serogroup D (n=78). Disk diffusion testing showed *Salmonella* serogroup A strains were susceptible to ampicillin, ceftriaxone, cefotaxime, ciprofloxacin and ceftazidime while other serogroups had resistance phenotypes (Table 2). As show in Table 2, all strains were susceptible to ciprofloxacin.

**Table 2.** Susceptibility of *Salmonella* serogroups to antibiotics

Antibiotics^*^	**Group A**	**Group B**	**Group C**	**Group D**	**Total**
AMP (10 μg)	-	2	6	3	11
CTX (30 μg)	-	-	4	2	6
CRO (30 μg)	-	6	14	20	40
CAZ (30 μg)	-	6	14	20	40
CIP (5 μg)	-	-	-	-	0
	n=1	n=18	n=41	n=78	n=138

* AMP, ampicillin; CRO, ceftriaxone; CTX, cefotaxime; CAZ, ceftazidime; CIP, ciprofloxacin

According to results of antibiotic susceptibility testing, four resistance profiles were obtained as follows: 1) AMP resistance, 2) CRO, CAZ resistance, 3) CRO, CAZ, AMP resistance and 4) CTX, CRO, CAZ, AMP, CIP resistance (Table 3).

**Table 3.** Antibiotic resistance profiles among *Salmonella* serogroups.

**Profiles number**	**Antibiotics resistance profile**	**Serogroup (number of isolates)**
1	AMP	B (1), C (1) and D (1)
2	CRO, CAZ	B (5), C (9) and D (17)
3	CRO, CAZ, AMP	B (1), C (2) and D (1)
4	CTX, CRO, CAZ, AMP	C (4) and D (2)

### ESBLs screening test.

**Fig. 1.**
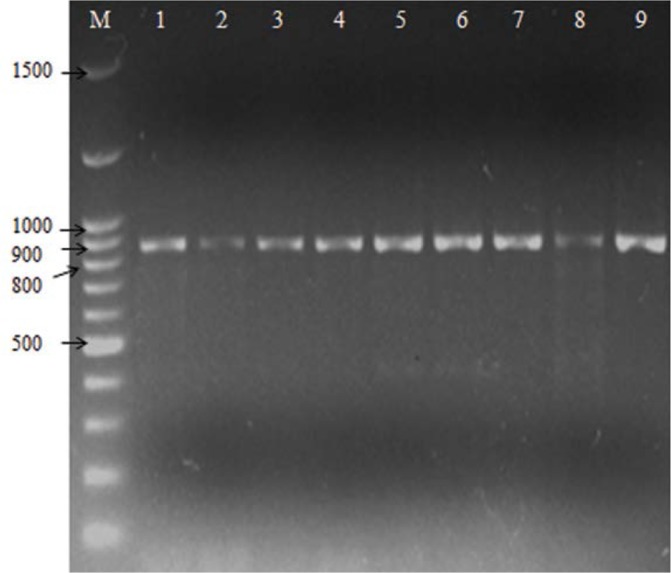
Electrophoresis of TEM amplicons (867 bp) on agarose gel 1%. Lane M, DNA marker, lane 1–9, PCR products from *Salmonella* isolates.

**Fig. 2.**
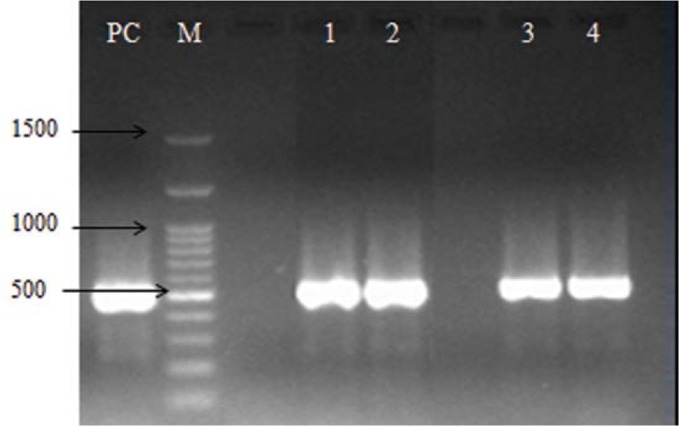
Electrophoresis of *bla*_CTX_ gene amplicons (519 bp) on agarose gel 1%. Lane PC, positive control; lane M, DNA marker; lane 1–4PCR, products from *Salmonella* isolates.

**Fig. 3.**
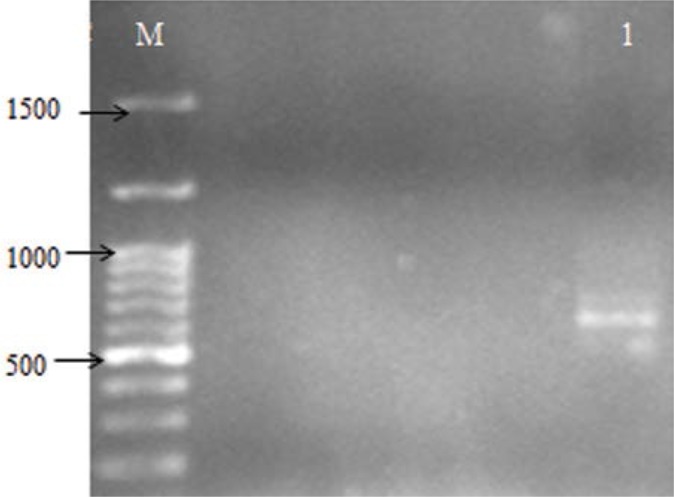
Electrophoresis of *bla*_SHV_ gene amplicons (7796 bp) on agarose gel 1%. Lane PC, positive control; lane M, DNA marker; lane 1–4 PCR, product from *Salmonella* isolate.

In this study, the *bla*_CTX_, *bla*_TEM_ and *bla*_SHV_ β-lactamse genes were detected among isolates using the double disc synergy method and also confirmed by PCR (Figs. 1–3). Forty strains (28.98%) of 138 isolates were positive for ESBL genes belonging to ESBL-CTX (n=17, 12.3%), ESBL-TEM (n=40, 29.9%) and ESBL-SHV (n=4, 2.89%) β-lactamase.

## DISCUSSION

Antibiotic resistance among *Salmonella* species is a major challenge in public health and is rapidly increasing (18). Recently, multidrug resistant strains of *Salmonella* have also been reported (19–23). These strains cause high mortality and morbidity and in most cases, leading to bloodstream infections and patient hospitalization (24). Multidrug resistant strains are common among animal populations and spreading worldwide (25). Although this phenotype has been reported in *S. enterica* serovar. Typhimurium, *S. enterica* serovar. Paratyphi and *S. enterica* serovar. Agona (26, 27), it may exist in other serotypes such as *S. enterica* serovar. Enteritidis (28, 29). Recently, resistance to ampicillin, chloramphenicol, trimethoprim/sulfamethoxazole, quinolones and cephalosporins have also been reported among *Salmonella* strains (30).

Cephalosporins are commonly used to treat *Salmonella* infections. However, in our study, resistance to cephalosporins was observed in two serogroups, indicating high prevalence of cephalosporins resistance among Iranian isolates. These results are similar to other studies reporting resistance to cephalosporins in *S. enterica* serovars isolated from other countries. Rotimi et al. found that from a total of 407 isolates, 116 isolates possessed the ESBL resistance phenotypes (12.1% CTX-M-15; 24.6% TEM (31). The high rates of multidrug resistance and ESBL positive *Salmonella* have also been observed in China. Yu et al. found that most *S. enterica* serovar. Typhimurium isolates had a resistance phenotype to multiple antimicrobial agents, including tetracycline, trimethoprim/sulfamethoxazole, ampicillin, chloramphenicol and cefotaxime and also a total of 79.0% of *S. enterica* serovar. Typhimurium isolates harbor *bla*_TEM-1b_ (32). According to above data and emergence of ESBL producing *Salmonellae* strains, the effectiveness of cephalosporins may be challengeable against *Salmonella* infections.

In present study, all isolates were found to be susceptible to ciprofloxacin. The result is inconsistent with other reports worldwide (33–35). Cui et al. showed that the high prevalence of resistance to fluoroquinolone among *S.* Typhimurium isolates may be affected by various factors such as hospitalization (36). Using livestock products is also another way of acquisition of ciprofloxacin-resistant *S.* Typhimurium for the reason that livestock products are a common source of salmonellosis (37). Susceptibility to ciprofloxacin among other strains such as *S. enterica* serovar. Typhi has also been identified (38). It seems that ciprofloxacin can be considered as the drug of choice in treating *Salmonella* infections in Iran (39).

In conclusion, our study showed high rates of ESBL positive-*Salmonella* strains isolated from pediatric patients, suggesting a necessity to substitute the cephalosporins with a proper alternative. Although all of isolates in our study were found to be susceptible to ciprofloxacin, it is better to perform antibiotic susceptibility testing before treatment of *Salmonella* infections.
